# Supplementary material for: Contributions of cortical neuron firing patterns, synaptic connectivity, and plasticity to task performance
Source: Nat Commun. 2024 Jul 17;15:6023. doi: 10.1038/s41467-024-49895-6 (PMC11255273; doi:10.1038/s41467-024-49895-6)
Supplement: Supplementary file 3 — Reporting Summary [file 41467_2024_49895_MOESM3_ESM.pdf]

nature portfolio

Corresponding author(s): Michele N. Insanally, Robert C. Froemke

Last updated by author(s): May 20, 2024

Reporting Summary

Nature Portfolio wishes to improve the reproducibility of the work that we publish. This form provides structure for consistency and transparency in reporting. For further information on Nature Portfolio policies, see our Editorial Policies and the Editorial Policy Checklist.

Statistics

For all statistical analyses, confirm that the following items are present in the figure legend, table legend, main text, or Methods section.

n/a	Confirmed	
☐	☒	The exact sample size (*n*) for each experimental group/condition, given as a discrete number and unit of measurement
☐	☒	A statement on whether measurements were taken from distinct samples or whether the same sample was measured repeatedly
☐	☒	The statistical test(s) used AND whether they are one- or two-sided<br>*Only common tests should be described solely by name; describe more complex techniques in the Methods section.*
☒	☐	A description of all covariates tested
☐	☒	A description of any assumptions or corrections, such as tests of normality and adjustment for multiple comparisons
☐	☒	A full description of the statistical parameters including central tendency (e.g. means) or other basic estimates (e.g. regression coefficient) AND variation (e.g. standard deviation) or associated estimates of uncertainty (e.g. confidence intervals)
☐	☒	For null hypothesis testing, the test statistic (e.g. *F*, *t*, *r*) with confidence intervals, effect sizes, degrees of freedom and *P* value noted<br>*Give P values as exact values whenever suitable.*
☒	☐	For Bayesian analysis, information on the choice of priors and Markov chain Monte Carlo settings
☒	☐	For hierarchical and complex designs, identification of the appropriate level for tests and full reporting of outcomes
☐	☒	Estimates of effect sizes (e.g. Cohen's *d*, Pearson's *r*), indicating how they were calculated

Our web collection on statistics for biologists contains articles on many of the points above.

Software and code

Policy information about availability of computer code

Data collection	All RNN simulations conducted in Julia 1.7.2 with standard packages and custom written code to run simulations (see GitHub repository at https://github.com/albannalab/InsanallyAlbanna2022). Extracellular recordings in rats made with a custom-programmed microcontroller (Med Associates) and spikes were sorted using Offline Sorter (Plexon Inc). For cell-attached and whole-cell recordings, behavioral events were monitored and controlled by custom-written programs in MATLAB that interfaced with an RZ6 processor (Tucker-Davis Technologies). Cell-attached and whole-cell recordings were collected using Clampex (v. 10.7; Molecular Devices)
Data analysis	Whole-cell data were analyzed with Clampfit 10 (Molecular Devices). All other analysis was completed using Python 3.7.9 with standard packages (including numpy 1.19.1, matplotlib 3.3.1, scipy 1.5.2, statsmodels 0.12.0) and custom written code to process data, conduct analyses, and generate plots (see GitHub repository at https://github.com/albannalab/InsanallyAlbanna2022)

For manuscripts utilizing custom algorithms or software that are central to the research but not yet described in published literature, software must be made available to editors and reviewers. We strongly encourage code deposition in a community repository (e.g. GitHub). See the Nature Portfolio guidelines for submitting code & software for further information.

Data

Policy information about availability of data

All manuscripts must include a data availability statement. This statement should provide the following information, where applicable:

- Accession codes, unique identifiers, or web links for publicly available datasets
- A description of any restrictions on data availability
- For clinical datasets or third party data, please ensure that the statement adheres to our policy

The data that support the findings of this study are available on Github (https://github.com/albannalab/InsanallyAlbanna2022) and from the corresponding authors upon reasonable request.

Research involving human participants, their data, or biological material

Policy information about studies with human participants or human data. See also policy information about sex, gender (identity/presentation), and sexual orientation and race, ethnicity and racism.

Reporting on sex and gender	n/a
Reporting on race, ethnicity, or other socially relevant groupings	n/a
Population characteristics	n/a
Recruitment	n/a
Ethics oversight	n/a

Note that full information on the approval of the study protocol must also be provided in the manuscript.

Field-specific reporting

Please select the one below that is the best fit for your research. If you are not sure, read the appropriate sections before making your selection.

☒ Life sciences ☐ Behavioural & social sciences ☐ Ecological, evolutionary & environmental sciences

For a reference copy of the document with all sections, see nature.com/documents/nr-reporting-summary-flat.pdf

Life sciences study design

All studies must disclose on these points even when the disclosure is negative.

Sample size	Power analysis were conducted to determine the number of experimental trials required as well as the number of RNN simulations . All power analyses were conducted with beta = 0.8 to determine sufficient sample size.
Data exclusions	For extracellular rat recordings, single-units were identified on each tetrode by manually classifying spikes projected as points in 2D or 3D feature space. Clustering quality was assessed based on the Isolation Distance and L ratio sorting quality metrics and artifacts were rejected based on refractory period violations (< 1 msec). For cell-attached mouse recordings, artifacts were manually rejected based on refractory period violations (< 1 msec) and waveform shape. For all recordings, recording sessions were truncated if significant non-stationarity was detected.
Replication	We ensured all experiments were successfully reproduced across animals and neuronal populations. Extracellular recordings in rats were replicated with N = 11 rats. The results from our whole-cell recordings were successfully reproduced by two electrophysiologists (co-authors on the manuscript) with N = 5 mice. All RNN simulations were conducted with multiple networks (typically at least N=8) initialized with random weight matrices.
Randomization	Randomization was not necessary for animal experiments because all animals underwent the same training protocol and all cells were included for analysis. All RNN simulations were initialized with random weight matrices as described in the methods.
Blinding	Because all animals underwent the same training procedures and cells were analyzed after data collection no blinding was required.

Reporting for specific materials, systems and methods

We require information from authors about some types of materials, experimental systems and methods used in many studies. Here, indicate whether each material, system or method listed is relevant to your study. If you are not sure if a list item applies to your research, read the appropriate section before selecting a response.

Materials & experimental systems

n/a	Involved in the study
☒	☐ Antibodies
☒	☐ Eukaryotic cell lines
☒	☐ Palaeontology and archaeology
☐	☒ Animals and other organisms
☒	☐ Clinical data
☒	☐ Dual use research of concern
☒	☐ Plants

Methods

n/a	Involved in the study
☒	☐ ChIP-seq
☒	☐ Flow cytometry
☒	☐ MRI-based neuroimaging

Animals and other research organisms

Policy information about studies involving animals; ARRIVE guidelines recommended for reporting animal research, and Sex and Gender in Research

Laboratory animals	2-3 month old adult rodents were used in all experiments. For single-unit recordings in freely moving rats, 11 adult Sprague-Dawley rats were used. For cell-attached recordings in head-fixed mice, 10 adult C57Bl/6 mice were used.
Wild animals	This study did not involve wild animals.
Reporting on sex	For single-unit recordings in freely moving rats, 5 adult male and 6 adult female Sprague-Dawley rats were used. For cell-attached recordings in head-fixed mice, 4 adult male and 6 adult female C57Bl/6 mice were used.
Field-collected samples	The study did not involve samples collected from the field.
Ethics oversight	All animal procedures were performed in accordance with National Institutes of Health standards and were conducted under a protocol approved by the New York University School of Medicine Institutional Animal Care and Use Committee.

Note that full information on the approval of the study protocol must also be provided in the manuscript.
